# Intra‐arterial gas, a clue for diagnosis of peri‐aortic inflammation due to infection

**DOI:** 10.1002/ccr3.3367

**Published:** 2020-09-20

**Authors:** Daisuke Omura, Masatoshi Ogata, Yoshio Sakane, Ryuichi Matsuo, Harushige Nakatsukasa, Hideharu Hagiya, Fumio Otsuka

**Affiliations:** ^1^ Department of General Medicine Okayama University Graduate School of Medicine, Dentistry and Pharmaceutical Sciences Okayama Japan; ^2^ Department of Internal Medicine Mizushima Central Hospital Kurashiki Japan

**Keywords:** aortic aneurysm, *Salmonella*

## Abstract

We have presented a case of Salmonella‐induced infective aortic aneurysm in which the presence of peri‐aortic gas was a clue for diagnosis. The disease is clinically infrequent but potentially has a high mortality rate. Clinicians should consider this fatal disease from any trivial findings.

## Graphical abstract

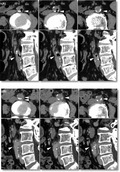

A 70‐year‐old diabetic woman was admitted to our hospital with a complaint of high fever accompanying shaking chills for 3 days. The patient's body temperature was increased to 39°C, and blood analysis showed elevations of leukocytes (17 400/µL) and C‐reactive protein (16.5 mg/dL). Computed tomography revealed air‐density spots in the aortic wall (Figure 1A), and *Salmonella enteritidis* serotype O9 was detected by blood culture. She had no other notable symptoms including digestive symptoms and had no history of eating raw food. Under a tentative diagnosis of Salmonella‐induced infective saccular aneurysm in the infrarenal abdominal aorta, the patient underwent antibiotic treatment with ampicillin/sulbactam. On day 11, the air had disappeared and the aortic wall showed a varicose deformity (Figure 1B). The patient recovered completely with 8‐week antibiotic therapy without any operation.

**FIGURE 1:**
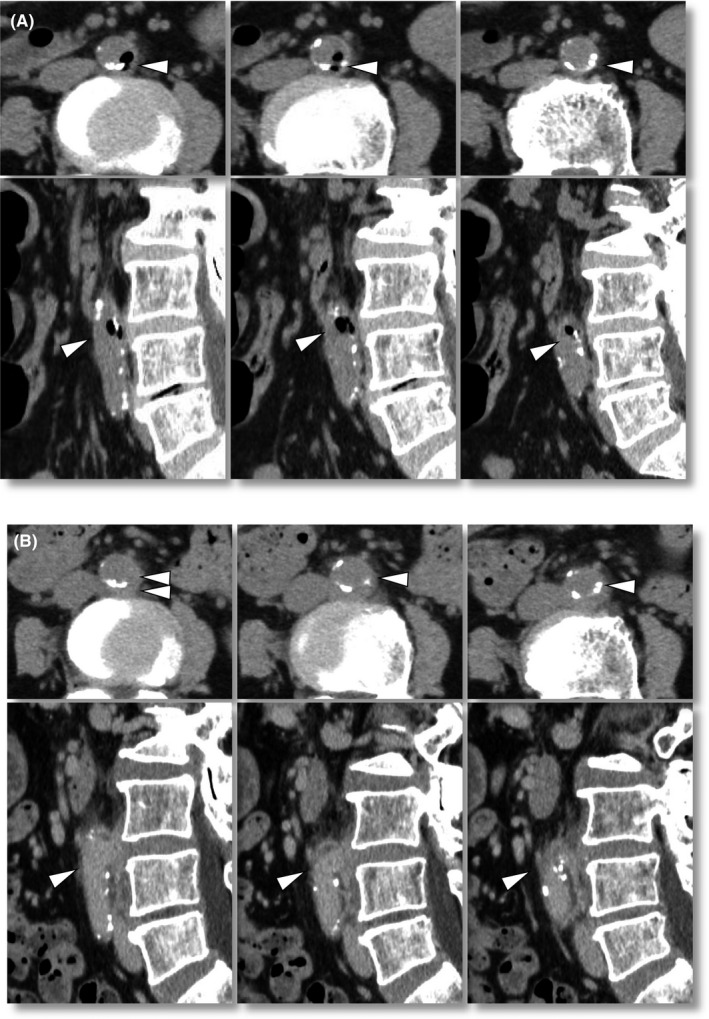
Abdominal computed tomography on admission (A) and on day 11 (B). Air‐density spots were found in the aortic wall (A, arrowheads). They later disappeared, and outpouching of the arterial wall emerged at the corresponding site (B)

Cases of infective aortic aneurysms account for only about 1%‐3% of total cases of aortic aneurysm. However, the case fatality has been reported to be as high as 18% in 2 years.^1^ *Salmonella* species, as detected in this case, are known as a common pathogen of crucial infection.^2^ Not only intra‐aortic gas but also the presence of a penetrating aortic ulcer suggests acute aortic syndrome, which requires urgent management.^3^ Without treatment, mycotic aneurysms are associated with high mortality from rupture or uncontrolled sepsis. Thus, clinicians should pay attention to these findings for early diagnosis of the disease.

## CONFLICT OF INTEREST

We have no financial relationships to disclose.

## AUTHOR CONTRIBUTIONS

DO and HN: wrote the first draft and managed all of the submission process. MO, YS, and RM: supervised clinical management of the patient. HH and FO: contributed to clinical management of the patient and revised the manuscript.
